# Induction of Paraptotic Cell Death in Breast Cancer Cells by a Novel Pyrazolo[3,4-*h*]quinoline Derivative through ROS Production and Endoplasmic Reticulum Stress

**DOI:** 10.3390/antiox11010117

**Published:** 2022-01-05

**Authors:** Phuong Linh Nguyen, Chang Hoon Lee, Heesoon Lee, Jungsook Cho

**Affiliations:** 1 Integrated Research Institute for Drug Development, College of Pharmacy, Dongguk University-Seoul, Goyang 10326, Korea; phuonglinh212126@gmail.com (P.L.N.); uatheone@dongguk.edu (C.H.L.); 2 College of Pharmacy, Chungbuk National University, Cheongju 28160, Korea; medchem@chungbuk.ac.kr

**Keywords:** paraptosis, pyrazolo[3,4-*h*]quinoline scaffold, breast cancer cells, drug resistance, cytoplasmic vacuolization, reactive oxygen species, endoplasmic reticulum stress

## Abstract

Chemotherapy has been a standard intervention for a variety of cancers to impede tumor growth, mainly by inducing apoptosis. However, development of resistance to this regimen has led to a growing interest and demand for drugs targeting alternative cell death modes, such as paraptosis. Here, we designed and synthesized a novel derivative of a pyrazolo[3,4-*h*]quinoline scaffold (YRL1091), evaluated its cytotoxic effect, and elucidated the underlying molecular mechanisms of cell death in MDA-MB-231 and MCF-7 breast cancer (BC) cells. We found that YRL1091 induced cytotoxicity in these cells with numerous cytoplasmic vacuoles, one of the distinct characteristics of paraptosis. YRL1091-treated BC cells displayed several other distinguishing features of paraptosis, excluding autophagy or apoptosis. Briefly, YRL1091-induced cell death was associated with upregulation of microtubule-associated protein 1 light chain 3B, downregulation of multifunctional adapter protein Alix, and activation of extracellular signal-regulated kinase 1/2 and c-Jun N-terminal kinase. Furthermore, the production of reactive oxygen species (ROS) and newly synthesized proteins were also observed, subsequently causing ubiquitinated protein accumulation and endoplasmic reticulum (ER) stress. Collectively, these results indicate that YRL1091 induces paraptosis in BC cells through ROS generation and ER stress. Therefore, YRL1091 can serve as a potential candidate for the development of a novel anticancer drug triggering paraptosis, which may provide benefit for the treatment of cancers resistant to conventional chemotherapy.

## 1. Introduction

According to the International Agency for Research on Cancer (https://gco.iarc.fr, accessed on 1 September 2021), breast cancer (BC) has overtaken lung cancer as the most frequently diagnosed cancer globally as of 2020, accounting for approximately 12% of all new cases [1]. The mortality of BC ranks first in women worldwide and is responsible for about 16% of cancer deaths [1]. BC is a heterogeneous disease comprising distinct subtypes with different responses and clinical outcomes [2]. Moreover, despite recent advances in early detection and treatment, acquired resistance to chemotherapy often leads to therapeutic failures in BC [3]. Therefore, there is a constant need for the discovery of novel potential anticancer agents that can address the challenges of heterogeneity and resistance in BC.

Most chemotherapy conventionally used in a standard anticancer regimen suppresses tumor growth mainly through induction of apoptosis. However, many cancer cells, probably due to their ever-evolving nature, acquire the capability to circumvent apoptotic cell death and develop resistance to conventional chemotherapy. Hence, there has been a growing interest in and demand for the discovery of drugs targeting alternative cell death modes other than apoptosis. Paraptosis is recently described as a form of programmed cell death (PCD), characterized by features distinct from apoptotic or autophagic cell death [4]. The hallmark of paraptosis is the formation of extensive cellular vacuolization, which is generated from either endoplasmic reticulum (ER) stress or mitochondria swelling [5]. Common molecular changes associated with paraptosis include upregulation of microtubule-associated protein 1 light chain 3B (LC3B), activation of mitogen-activated protein kinase family members (MAPKs), including extracellular signal-regulated kinase 1/2 (ERK1/2), c-Jun N-terminal kinase (JNK), and p38 MAPK, and downregulation of multifunctional adapter protein Alix [6,7]. It is also shown to require new protein synthesis or reactive oxygen species (ROS) [6,7]. With biochemical characteristics distinct from apoptosis, paraptotic cell death may be targeted by novel candidates, providing a rational therapeutic strategy for the treatment of cancers resistant to conventional chemotherapy. Several natural compounds, such as curcumin [8], celastrol [9], and withaferin A, [10] have been reported to induce paraptosis in BC cells. In addition, synthetic derivatives, such as pyrazinoylguanidine compounds [11] and copper and zinc 2-(pyridin-2-yl)imidazo[1,2-*a*]pyridine complexes [12], are demonstrated to induce paraptosis in BC cells.

Pyrazoloquinolines are becoming an attractive scaffold in medicinal chemistry owing to their remarkable biological activities, such as anticancer, anti-anxiety, antibacterial and antiviral [13]. For instance, pyrazolo[3,4-*h*]quinolines and pyrazolo[4,3-*f*]quinolines [14,15] act as promising photosensitizing agents and haspin kinase inhibitors in the treatment of cancer, respectively. In addition, the chromene moiety is an important structural element in a variety of natural and synthetic pharmacologically active compounds [16,17]. In this context, we have designed and synthesized angularly fused heterocyclic compounds as part of our continuing efforts to develop potential anticancer agents with heterocyclic scaffolds [18,19,20]. Among the synthesized compounds, 12-methyl-8,12-dihydrobenzo[5,6]chromeno[4,3-*b*]pyrazolo[3,4-*h*]quinoline (YRL1091) was found to exhibit cytotoxicity with extensive cytoplasmic vacuolization, a typical feature of paraptosis, in BC cells. This observation prompted us to investigate the molecular mechanisms of cell death underlying the cytotoxic effect of this compound.

In this study, considering the heterogeneity of BC, we employed two different types of human BC cell lines, MDA-MB-231 and MCF-7 cells. Although these two cell lines share some properties of BC, they have distinct characteristics, showing differential responses to anticancer therapies. MDA-MB-231 cells are a triple-negative BC (TNBC) cell line, lacking the expression of estrogen receptor, progesterone receptor, and human epidermal growth factor receptor 2. TNBC is characterized by highly aggressive, invasive, and poorly differentiated properties [21]. The lack of the estrogen receptor has rendered these cells insensitive to anti-estrogen therapy [22]. By contrast, MCF-7 cells expressing both estrogen and progesterone receptors are highly responsive to tamoxifen therapy [23]. Therefore, these two cell lines are widely used as in vitro cellular models representing invasive human BC with distinct characteristics. We also conducted our study using these two BC cell lines to examine whether YRL1091 exerted any differential biological actions in these cells. Overall, we aimed to investigate the anticancer effects of a novel pyrazoloquinoline derivative, YRL1091, and to elucidate its underlying molecular mechanisms of cell death in MDA-MB-231 and MCF-7 cells.

## 2. Materials and Methods

### 2.1. Synthesis of YRL1091

**Scheme 1:**
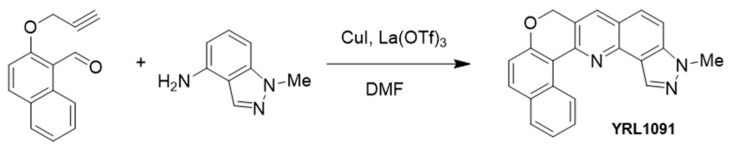
Synthesis of 12-methyl-8,12-dihydrobenzo[5,6]chromeno[4,3-*b*]pyrazolo[3,4-*h*]quinoline (YRL1091).

YRL1091 was synthesized according to the reported procedure [20]. In brief, 1.0 equivalent of 1-methyl-1H-indazol-4-amine was treated with 1.5 equivalents of 2-(prop-2-yn-1-yloxy)-1-naphthaldehyde in the presence of CuI (10 mol%) and La(OTf)_3_ (10 mol%) in dimethylformamide. The reaction mixture was heated at 173 °C for 2 h in a microwave reactor to obtain YRL1091 with a purity of more than 97%. It is a yellow solid compound, easily soluble in organic solvents like dimethyl sulfoxide (DMSO), and has a relative molecular mass of 338. Its structure was confirmed by nuclear magnetic resonance and is shown in Scheme 1.

For all our experiments, YRL1091 was prepared in DMSO to obtain a stock solution of 10 mM.

### 2.2. Chemicals and Reagents

Acridine orange (AO), anti-β-actin antibody (1:2000, cat#A5316), chloroquine (CQ), cycloheximide (CHX), 2ʹ,7ʹ-dichlorofluorescin diacetate (DCFH-DA), 3-methyladenine (3-MA), *N*-acetyl cysteine (NAC), rapamycin (Rapa), staurosporine (STS), sulforhodamine B (SRB), and trichloroacetic acid (TCA) were purchased from Sigma-Aldrich (St. Louis, MO, USA). Pan caspase inhibitor z-VAD-FMK (z-VAD) was bought from R&D Systems, Inc. (Minneapolis, MN, USA). Alexa Fluor 488-conjugated anti-mouse immunoglobulin G (IgG) and 4′,6-diamidino-2-phenylindole dihydrochloride (DAPI) were obtained from Thermo Scientific (Rockford, IL, USA). Dulbecco’s modified Eagle medium (DMEM) and fetal bovine serum (FBS) were supplied from Corning, Inc. (Corning, NY, USA). SP600125 was bought from Calbiochem (Darmstadt, Germany). U0126 and antibodies specifically recognizing Alix (1:1000, cat#2171), ATF4 (1:1000, cat#11815), binding immunoglobulin protein (BiP, 1:1000, cat#3177), calnexin (1:1000, cat#2433), cleaved caspase 3 (1:1000, cat#9661), cleaved caspase 9 (1:1000, cat#9505), caspase 3 (1:1000, cat#9665), LC3B (1:1000, cat#3868), CCAAT-enhancer-binding protein homologous protein (CHOP, 1:1000, cat#2895), poly-ADP-ribose polymerase (PARP, 1:1000, cat#9542), p62 (1:1000, cat#5114), non-phosphorylated ERK1/2 (1:1000, cat#4696), JNK (1:1000, cat#9252), and p38 MAPK (1:1000, cat#9212) or phosphorylated ERK1/2 (1:1000, cat#4377), JNK(1:1000, cat#4668), and p38 MAPK (1:1000, cat#9216), horseradish peroxidase (HRP)-conjugated rabbit (1:2000, cat#7074) and mouse IgG (1:2000, cat#7076), and ubiquitinated proteins (1:1000, cat#3936) were obtained from Cell Signaling Technology (Danvers, MA, USA). LC3B small interfering RNA (siRNA) and control siRNA were provided by Santa Cruz Biotechnology, Inc. (Dallas, TX, USA).

### 2.3. Cell Culture

The human BC cell lines including MDA-MB-231 and MCF-7 cells were supplied by the American Type Culture Collection (Manassas, VA, USA). These cells were maintained in DMEM containing 10% FBS and 1% antibiotic–antimycotic (final concentrations of 100 U/mL penicillin and 100 µg/mL streptomycin) in a humidified incubator at 37 °C with 5% CO_2_, as previously described [24,25].

### 2.4. Cell Viability

Cell viability was determined by SRB assay, which is used to examine the protein content of cells as described previously [26]. In brief, MDA-MB-231 and MCF-7 cells were seeded at a density of 1 × 10^4^ cells/well and 2 × 10^4^ cells/well, respectively, in sterile 96-well plates (100 μL/well) and grown overnight to 40–50% confluence at 37 °C with 5% CO_2_. After incubation, 100 μL of YRL1091 prepared in culture media was added to each well to furnish the working concentrations of 1, 3, 10, 30, 100, and 300 μM. Simultaneously, an equivalent volume of the vehicle (DMSO) was treated to the control cells. After the desired treatment time, the cells were fixed by gentle layering of cold 50% (w/v) TCA (50 μL/well, to 10% final concentration). The plates were incubated at 4 °C for 1 h, then washed three times with distilled water. The fixed cells were stained with 0.4% (w/v) SRB dissolved in 1% (v/v) acetic acid (50 μL/well) for 1 h at room temperature. Excess unbound dye was removed by washing with 1% acetic acid three times and the protein-bound dye was extracted with 10 mM Tris base solution (pH 10.5). The absorbance was measured at 510 nm using a microplate reader (SpectraMax M2e, Molecular Devices, Sunnyvale, CA, USA). Cell viability was expressed as the percentage of absorbance determined for vehicle-treated control cells.

### 2.5. Cell Morphology

To evaluate the changes in cell morphology, phase-contrast images were captured using a Nikon phase-contrast microscope (Nikon Instruments, Inc., Melville, NY, USA).

### 2.6. Measurement of ROS

Intracellular ROS production induced by YRL1091 treatment was assessed by DCFH-DA staining, as described previously with minor modifications [27,28]. MDA-MB-231 and MCF-7 cells were seeded into 24-well plates at a density of 1 × 10^5^ cells/well and 1.2 × 10^5^ cells/well, respectively, to achieve 70–80% confluence. Afterwards, the cells were treated with various concentrations of YRL1091 in the presence or absence of NAC for 12 h. The culture media were then replaced with phosphate-buffered saline (PBS) containing DCFH-DA at a final concentration of 10 µM and incubated in the dark at 37 °C for 30 min. After washing with PBS, the levels of intracellular ROS were quantitated based on the fluorescence detection of dichlorofluorescein as an oxidized product of DCFH using a microplate reader (SpectraMax M2e, Molecular Devices) with an excitation wavelength of 490 nm and emission wavelength of 520 nm. The levels of ROS production were expressed as percentages of the vehicle-treated control cells. The fluorescent signals were visualized using a fluorescence microscope (Nikon Instruments Inc.).

### 2.7. Transfection of Cells with siRNA for LC3B

MDA-MB-231 and MCF-7 cells were seeded in 60-mm culture dishes at a density of 1 × 10^6^ cells/well and 1.2 × 10^6^ cells/well, respectively, and incubated at 37 °C overnight to 75–80% confluence. Then, siRNA control and siRNA LC3B were transfected to MDA-MB-231 and MCF-7 cells using Lipofectamine 2000 transfection reagent (Invitrogen, Rockford, IL, USA) following the manufacturer’s instructions and as previously reported [24]. After 6 h of transfection, media containing siRNAs were replaced with new culture media, and cells were plated for further experiments including western blotting, SRB assay, and visualization of cell morphology.

### 2.8. Western Blotting

MDA-MB-231 and MCF-7 cells were seeded at a density of 4 × 10^5^ cells/well and 5 × 10^5^ cells/well, respectively, into 6-well plates and incubated overnight until 70–80% confluence. After the desired treatment, cells were washed with cold PBS and lysed with lysis buffer on ice for 30 min, as reported previously [28]. The protein concentrations were measured by a Bio-Rad DC protein assay kit (Bio-Rad Laboratories, Hercules, CA, USA).

Western blotting was performed as previously reported [28,29]. Briefly, the supernatants containing equivalent amounts of protein were boiled in loading buffer, separated by sodium dodecyl sulfate-polyacrylamide gel electrophoresis, and transferred to nitrocellulose membranes at 100 V for 90 min. The transferred membranes were blocked with 5% (w/v) non-fat dried skim milk (BD Biosciences, San Jose, CA, USA) in Tris-buffered saline containing 0.1% (v/v) Tween 20 (TBS-T) at room temperature for 60 min and incubated with primary antibodies in bovine serum albumin (MP Biomedicals, Solon, OH, USA) at 4 °C overnight. After washing with TBS-T, the membranes were incubated with HRP-conjugated secondary antibodies at room temperature for 120 min. Finally, specific bands were visualized with a Bio-Rad ChemiDoc XRS imaging system using enhanced chemiluminescent reagents (Bio-Rad Laboratories).

### 2.9. Immunofluorescence and Lysosomal Staining

To detect LC3B and ER-bound protein calnexin, immunofluorescence staining was conducted, as previously described [29]. Briefly, MDA-MB-231 and MCF-7 cells were seeded on coverslips placed in the wells of 24-well plates at a density of 5 × 10^4^ cells/well and 6 × 10^4^ cells/well, respectively. After YRL1091 treatment, the cells were fixed with 4% (v/v) paraformaldehyde for 15 min and permeabilized using 1% (v/v) Triton X-100 in PBS for 5 min. Nonspecific binding was then blocked with 5% (v/v) goat serum in PBS for 30 min. All coverslips were washed gently three times between each step. Afterward, the cells were incubated with anti-LC3B or anti-calnexin antibody at a 1:250 dilution overnight at 4 °C, followed by incubation with Alexa Fluor 488-anti-rabbit IgG secondary antibody at a 1:400 dilution in the dark at room temperature for 1 h. Coverslips with stained cells were mounted in an antifade mounting medium with DAPI. Fluorescent signals were detected using a Nikon confocal laser-scanning microscope (Nikon Instruments, Inc.).

To detect acidic vacuoles like lysosomes, lysosomal staining using AO was performed. MDA-MB-231 and MCF-7 cells were seeded at a density of 5 × 10^4^ cells/well and 6 × 10^4^ cells/well, respectively, into 24-well plates, incubated overnight to reach 70–80% confluence, and treated with YRL1091 or Rapa for 12 h. The cells were then stained with 10 μM AO in PBS for 20 min at 37 °C, followed by washing gently with PBS. Fluorescent images were captured using a fluorescence microscope (Leica DM2500, Leica Microsystems Ltd., Wetzlar, Germany).

### 2.10. Measurement of Apoptosis by Annexin V-FITC/PI Staining

To detect and quantify apoptotic cells, flow cytometry analysis was performed using a Dead Cell Apoptosis Kit with annexin V and PI (Thermo Scientific), according to the manufacturer’s instruction. In brief, MDA-MB-231 and MCF-7 cells were seeded in 6-well plates at a density of 4 × 10^5^ cells/well and 5 × 10^5^ cells/well, respectively, incubated overnight until 70–80% confluence, and treated with YRL1091 for 24 h or STS for 12 h. Both floating and adherent cells were harvested, washed with PBS two times, and re-suspended in annexin-binding buffer. Cells were then incubated with annexin and PI for 15 min at room temperature in the dark. Apoptotic analysis was conducted by the BD FACSAria™ III cell analyzer (BD Biosciences, CA, USA). The percentage of apoptotic cells was determined as the total rate of apoptotic cells in early-stage (Annexin/PI: +/−) and late-stage (Annexin/PI: +/+).

### 2.11. Statistical Analyses

All data were presented as the mean ± SEM from at least three independent experiments. Comparisons were tested by one-way analysis of variance (ANOVA) followed by Tukey’s test (SigmaPlot 12.5 software, Systat Software, San Jose, CA, USA). For all tests, *p* < 0.05 was considered statistically significant. IC_50_ values were determined by nonlinear regression using GraphPad Prism 5 (GraphPad software, Inc., La Jolla, CA, USA).

## 3. Results

### 3.1. YRL1091 Induces Cytotoxicity and Cytoplasmic Vacuolization in BC Cells

**Figure 1:**
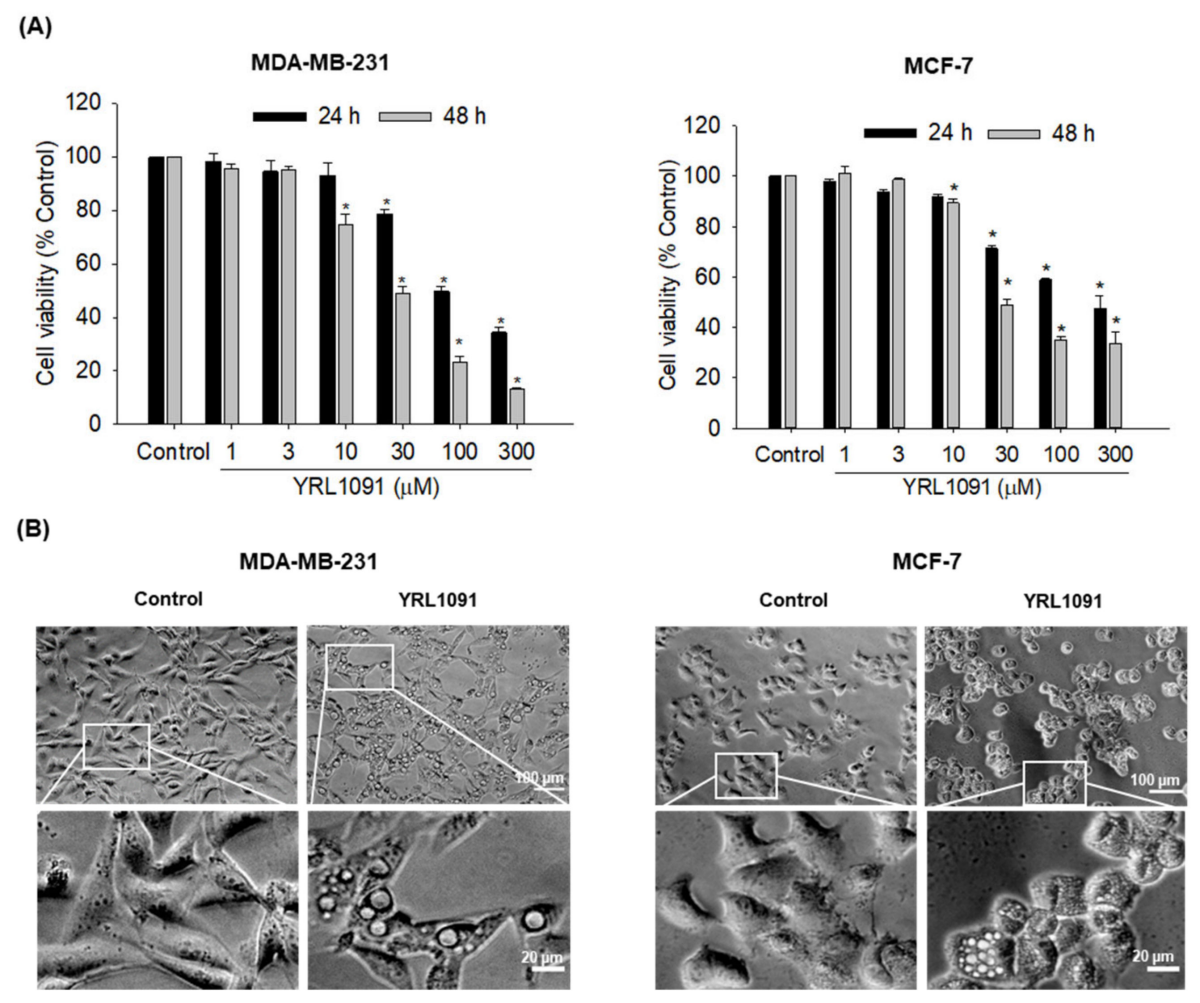
Induction of cytotoxicity and cytoplasmic vacuolization by YRL1091 in MDA-MB-231 and MCF-7 cells. (**A**) Cells were exposed to various concentrations (1–300 µM) of YRL1091 for 24 and 48 h. Cell viability was measured by SRB assay, as described in Materials and Methods section. The cell viability of each treated group was expressed as the percentage of that in control group and presented as the mean ± SEM from at least three independent experiments. * *p* < 0.05 vs. the vehicle control. (**B**) Cells were treated with 30 µM YRL1091 for 24 h, and the control cells were treated with DMSO instead. Changes in cell morphology were visualized using a phase-contrast microscope. Scale bar: 20 μm. Magnified images of white boxes are also provided.

We first examined the cytotoxic effect of YRL1091 in MDA-MB-231 and MCF-7 cells. The cells were treated with various concentrations of YRL1091 for 24 and 48 h, and cell viability was measured by SRB assay. YRL1091 significantly suppressed cell viability in both concentration- and time-dependent manners in MDA-MB-231 and MCF-7 cells (Figure 1A). Exposure of these cells to YRL1091 for 48 h yielded IC_50_ values of 28.9 ± 1.1 and 29.8 ± 1.0 µM, respectively. We observed the morphological changes in these cells under a phase-contrast microscope after YRL1091 exposure. Numerous vacuoles were observed in the cytoplasm of both MDA-MB-231 and MCF-7 cells treated with YRL1091 at the concentration of 30 μM for 24 h (Figure 1B). The formation of cytoplasmic vacuolization often accompanies cell death [5]. We supposed that the cytotoxic effect of YRL1091 on BC cells was associated with the occurrence of cytoplasmic vacuolization and further investigated the underlying mechanisms of cell death caused by YRL1091.

### 3.2. YRL1091-Induced Cell Vacuolization Shows the Features of Paraptosis

#### 3.2.1. Role of LC3B in YRL1091-Induced Cytoplasmic Vacuolization and Cell Death

**Figure 2:**
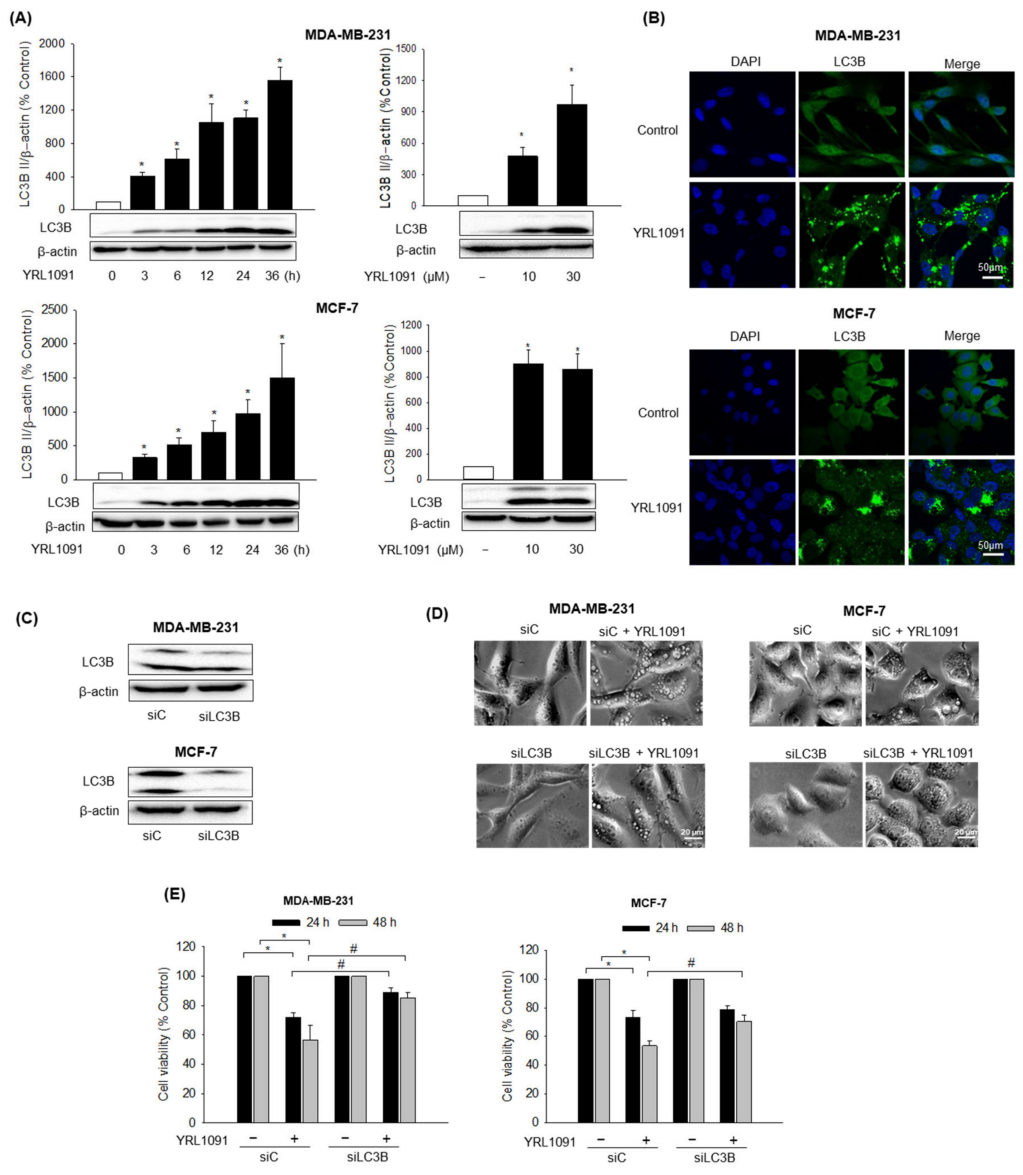
Role of LC3B in YRL1091-induced cytoplasmic vacuolization and cell death in MDA-MB-231 and MCF-7 cells. (**A**) Cells were challenged with 30 µM YRL1091 for the indicated times (left) or 24 h at the indicated concentrations (right). The levels of LC3B expression were measured by western blotting, as described in Materials and Methods. β-Actin was used for normalization. Each data point was calculated as the percentage of protein level in vehicle-treated control cells. Representative bands from at least three independent experiments were shown. (**B**) Cells were treated with 30 µM YRL1091 for 12 h and then stained with anti-LC3B antibody. Immunofluorescence images were obtained as described in Materials and Methods. Scale bar: 50 µm. (**C**–**E**) Cells were transfected with either siC or siLC3B as indicated in Materials and Methods. (**C**) The levels of LC3B were analyzed by western blotting. (**D**) Transfected cells were treated with YRL1091 (30 µM) for 12 h, and cell morphology was visualized using a phase-contrast microscope. Scale bar: 20 µm. (**E**) Transfected cells were exposed to YRL1091 (30 µM) for either 24 or 48 h and cell viability was measured by SRB assay. The cell viability of each treated group was expressed as the percentage of that in control group. Data are presented as the mean ± SEM from at least three independent experiments. * *p* < 0.05 vs. vehicle-treated control cells; # *p* < 0.05 vs. YRL1091-treated siC cells. C, Control.

One of the key characteristics of paraptosis is the induction of cytoplasmic vacuolization. Therefore, we studied whether YRL1091-triggered cell death was paraptotic. It is well documented that LC3 protein plays a critical role in the formation of cytoplasmic vacuoles mediating paraptotic cell death [10,30,31]. Intriguingly, out of three isoforms of MAP1 LC3, only LC3B was shown to mediate cytoplasmic vacuolization [32]. Therefore, the levels of LC3B in MDA-MB-231 and MCF-7 cells in the presence of YRL1091 were examined. The cells were treated with YRL1091 at indicated concentrations (10 and 30 μM) for the indicated times (3–36 h), and the expression levels of LC3B were accessed by western blotting. The levels of LC3B expression were significantly elevated at the lowest concentration and earliest time point and continued to increase time- and concentration-dependently in both cell types, suggesting the involvement of LC3B in the induction of paraptosis (Figure 2A). The immunofluorescence analysis also revealed a higher intensity of LC3B protein in the YRL1091-treated cells than in the control-treated cells, validating the upregulation of LC3B (Figure 2B). To confirm association between the elevated expression of LC3B and cytoplasmic vacuolization-related cell death by YRL1091, the cells were transfected with siRNA targeting LC3B to knockdown the protein (Figure 2C). LC3B knockdown cells treated with YRL1091 showed decreased vacuole formation, compared to control siRNA-transfected cells (Figure 2D). In addition, the depletion of LC3B significantly reversed the cytotoxic effect of YRL1091 in MDA-MB-231 cells at 24 and 48 h (Figure 2E). Interestingly, the cytotoxic effect of YRL1091 was significantly reversed by LC3B knockdown in MCF-7 cells only at 48 h (Figure 2E). These data implicate LC3B as a key regulator of YRL1091-induced cytoplasmic vacuolization, which eventually leads to paraptosis in BC cells.

#### 3.2.2. Role of MAPKs in YRL1091-Induced Cytoplasmic Vacuolization and Cell Death

**Figure 3:**
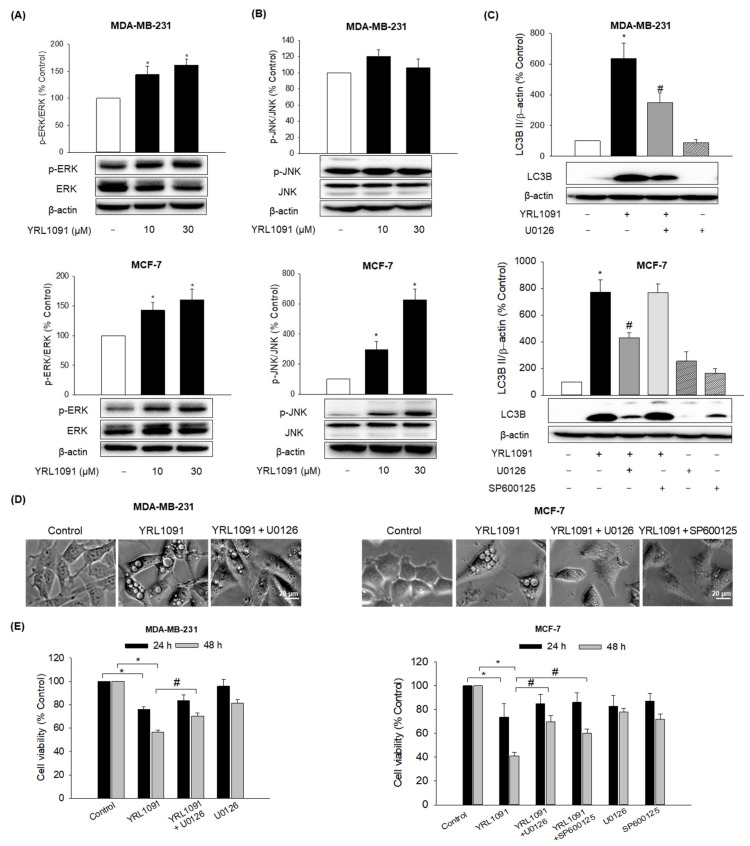
Roles of MAPKs in YRL1091-induced vacuolated cells and cell death in MDA-MB-231 and MCF-7 cells. (**A**,**B**) Cells were treated with YRL1091 (10 and 30 µM) for 24 h. (**A**) The phosphorylated ERK1/2 and (**B**) JNK were determined by western blotting. β-Actin was used for normalization. (**C**–**E**) Cells were pretreated for 2 h with ERK and JNK inhibitors (U0126 and SP600125, respectively, at 20 µM) and then YRL1091 (30 µM) was added. (**C**) The levels of LC3B protein were measured after 24 h of treatment using western blotting. Each data point was calculated as the percentage of protein level in vehicle-treated control cells. Representative blots from at least three independent experiments were displayed. (**D**) Cell morphology after 12 h of treatment was visualized using a phase-contrast microscope. Scale bar: 20 µm. (**E**) Cell viability at 24 or 48 h of treatment was examined by SRB assay. The cell viability of each treated group was expressed as the percentage of that in control group. Data are presented as the mean ± SEM from at least three independent experiments. * *p* < 0.05 vs. vehicle-treated control cells; # *p* < 0.05 vs. YRL1091-treated cells.

The involvement of MAPK family members including ERK1/2, JNK, and p38 MAPK in paraptosis has been demonstrated in a vast literature [33]. We next evaluated the effect of YRL1091 on the activation of MAPKs in MDA-MB-231 and MCF-7 cells. The cells were exposed to the indicated concentrations of YRL1091 for 24 h, and MAPK activation was assessed by measuring the phosphorylated forms of ERK1/2, JNK, and p38 MAPK. The phosphorylated ERK1/2 was significantly enhanced by YRL1091 treatment in both cell types at 10 µM and above (Figure 3A). By contrast, YRL1091 unambiguously accentuated the phosphorylation of JNK in MCF-7 cells but not in MDA-MB-231 cells (Figure 3B). There were no changes in the phosphorylation of p38 MAPK in both cell types (data not shown).

We then examined the effects of specific MAPK inhibitors in YRL1091-treated BC cells. U0126, an ERK1/2 inhibitor, remarkably reduced the elevated expressions of LC3B (Figure 3C), as well as the numbers of vacuolated cells (Figure 3D), subsequently diminishing YRL1091-induced cell death in MDA-MB-231 and MCF-7 cells at 48 h (Figure 3E). SP600125, a specific JNK inhibitor, did not affect the increased protein level of LC3B in YRL1091-treated MCF-7 cells (Figure 3C); however, it reversed the occurrence of vacuolated cells (Figure 3D) and significantly blocked YRL1091-induced cell death in MCF-7 cells at 48 h (Figure 3E). Therefore, ERK pathway positively regulates YRL1091-induced paraptotic cell death in both MDA-MB-231 and MCF-7 cells, while JNK pathway may be involved in the formation of cytoplasmic vacuolization and cell death in MCF-7 cells without altering LC3B expression.

#### 3.2.3. Effect of YRL1091 on the Expression of Alix Protein

**Figure 4:**
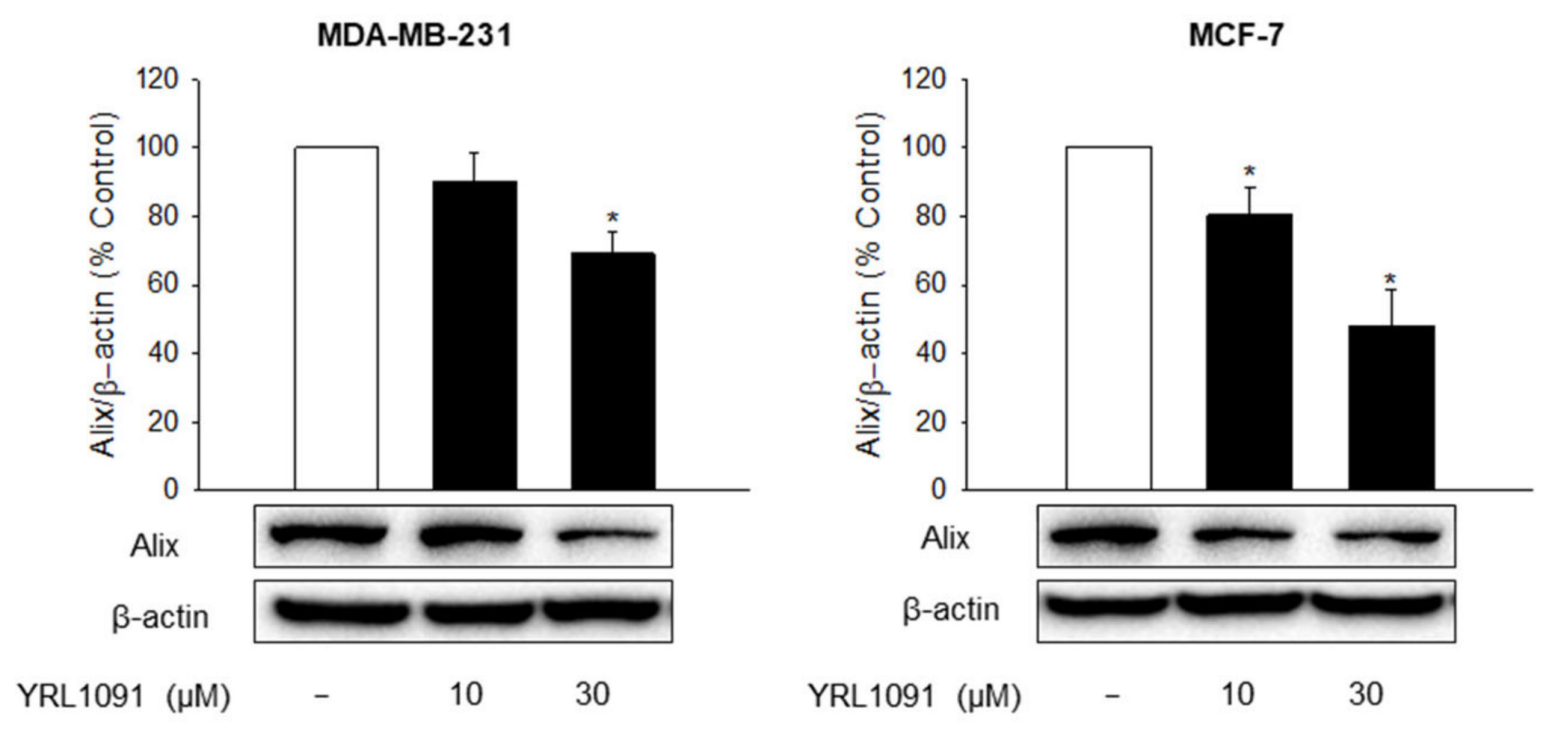
Effect of YRL1091 on the expression of Alix protein in MDA-MB-231 and MCF-7 cells. Cells were treated with YRL1091 at 10 and 30 μM for 24 h. The levels of Alix were measured by western blotting. Each data point was calculated as the percentage of protein level in vehicle-treated control cells. Representative bands from at least three independent experiments were shown. * *p* < 0.05 vs. vehicle-treated control cells.

The downregulation of AIP-1/Alix protein is also a typical feature of paraptosis that distinguishes it from apoptosis [33]. Thus, the expression of Alix was measured by western blotting after YRL1091 treatment for 24 h. The exposure of MDA-MB-231 and MCF-7 cells to YRL1091 drastically decreased the level of Alix expression (Figure 4), providing further evidence for paraptosis-mediated cell death in these cells.

#### 3.2.4. Effect of YRL1091 on the Activation of Caspase 9

**Figure 5:**
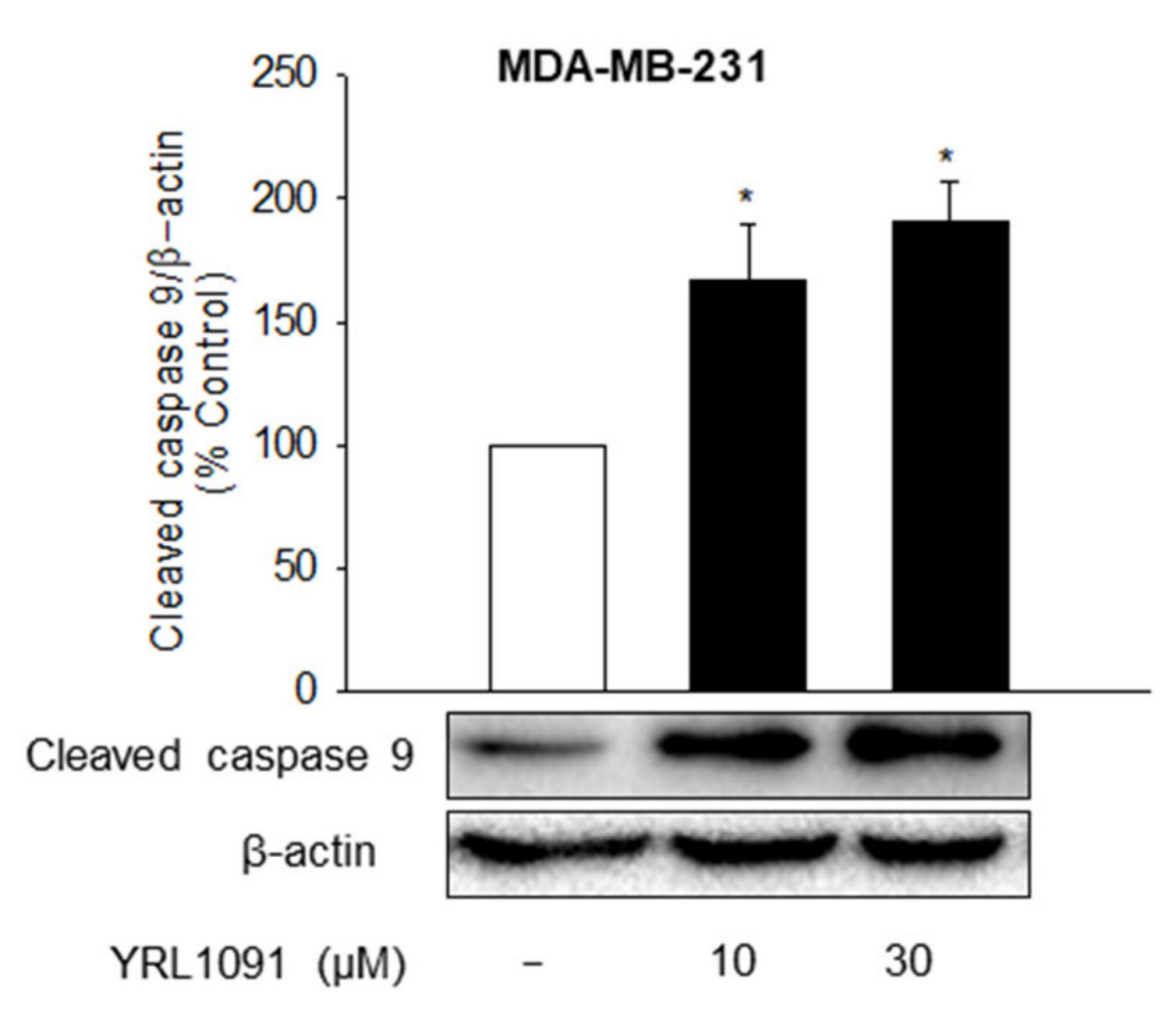
Effect of YRL1091 on the activation of caspase 9 in MDA-MB-231 cells. Cells were treated with YRL1091 at 10 and 30 μM for 24 h. The level of cleaved caspase 9 was measured by western blotting. Each data point was calculated as the percentage of protein level in vehicle-treated control cells. Representative bands from at least three independent experiments were shown. * *p* < 0.05 vs. vehicle-treated control cells.

Although paraptosis is a caspase-independent PCD, caspase 9 activation is considered a probable mechanism of paraptosis [7]. Therefore, we evaluated the effect of YRL1091 on the activation of caspase 9 in both types of BC cells. There was a dramatic augmentation of cleaved caspase 9 by YRL1091 in MDA-MB-231 cells (Figure 5). Hence, paraptosis in YRL1091-treated MDA-MB-231 cells may require caspase 9 activation unless apoptosis is also involved in YRL1091-induced cell death. By contrast, however, no bands corresponding to the cleaved caspase 9 were detected in MCF-7 cells (data not shown).

### 3.3. YRL1091-Mediated Cytoplasmic Vacuolization and Cell Death Show Non-Autophagic and Non-Apoptotic Characteristics

#### 3.3.1. Non-Autophagic Characteristics

**Figure 6:**
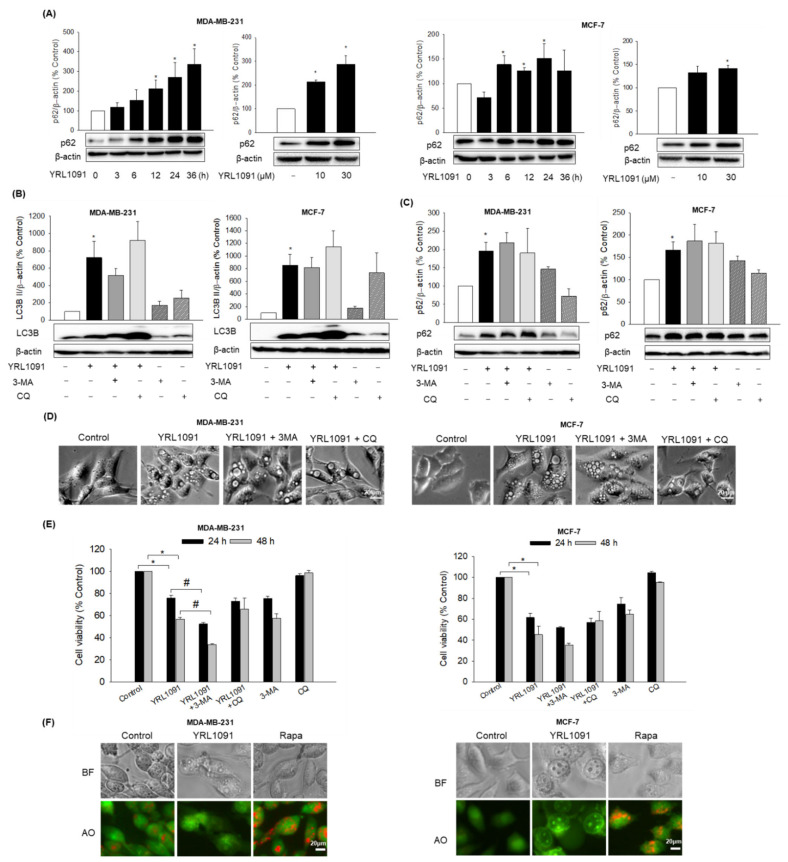
Autophagy-independent features of YRL1091-treated MDA-MB-231 and MCF-7 cells. (**A**) Cells were challenged with 30 µM YRL1091 for various times (left) or 24 h at the indicated concentrations (right). Levels of p62 were determined by western blotting. (**B**–**E**) Cells were treated with 30 µM YRL1091 in the presence or absence of 3-MA (5 mM, pretreatment for 2 h) or CQ (50 µM, 5 h before cell lysis). Levels of (**B**) LC3B and (**C**) p62 were measured by western blotting. Each data point was calculated as the percentage of protein level in vehicle-treated control cells. Representative blots from at least three independent experiments were shown. (**D**) Vacuolated cells were observed under a microscope. Scale bar: 20 µm. (**E**) Cell viability at 24 and 48 h was examined by SRB assay. The cell viability of each treated group was expressed as the percentage of that in control group. Data are determined as the mean ± SEM from at least three independent experiments. * *p* < 0.05 vs. vehicle-treated control cells; # *p* < 0.05 vs. YRL1091-treated cells. (**F**) Cells were exposed to YRL1091 (30 µM, 12 h) or Rapa (1 µM, 6 h), and AO staining was carried out as described in the Materials and Methods. Bright-field (BF) and fluorescent images were provided. Scale bar: 20 µm.

The accumulation of autophagic vacuoles is a morphological characteristic of autophagic cell death [34]. In this context, we determined whether the cytotoxic effect of YRL1091 in BC cells was contributed by autophagy. If the key feature of the early stage of autophagy is the occurrence of autophagosomes owing to the hydrolysis of LC3 I to LC3 II, lysosomal degradation with the participation of the ubiquitin-binding protein p62 is the main process in the later stage of autophagy [35]. Each stage can be differentiated by monitoring specific autophagy markers. Thus, we tested whether YRL1091 triggered any features associated with autophagy. Rather than a gradual decrease of p62 as in autophagy, the expression of p62 was significantly increased by YRL1091 in time- and concentration-dependent fashions in both types of BC cells (Figure 6A). Additionally, to find the correlation of YRL1091-induced cell death with autophagy, if any, we interrogated the occurrence of alterations in LC3B and p62 proteins in response to two autophagy inhibitors having distinct mechanisms: 3-MA, an inhibitor of autophagosome formation, and CQ, an inhibitor of fusion of autophagosomes to lysosomes. The levels of relevant protein markers, cell morphology, and cell viability were measured after the cells were exposed to YRL1091 in the presence or absence of 3-MA or CQ. Either 3-MA or CQ failed to suppress the upregulated expressions of LC3B (Figure 6B) and p62 (Figure 6C) by YRL1091 treatment in BC cells, indicating that the changes in these proteins by YRL1091 did not involve autophagy. Moreover, the cells exposed to YRL1091 continued to be vacuolated even in the presence of the two different autophagy inhibitors (Figure 6D). The treatment with 3-MA or CQ did not reverse the cytotoxic effect of YRL1091 (Figure 6E). Interestingly, 3-MA rather augmented the cytotoxic effect of YRL1091 in MDA-MB-231 cells, whereas no significant change in its effect was observed in MCF-7 cells.

The vacuoles like lysosomes in autophagy are usually acidic in nature, and AO is known to emit an orange fluorescence in the acidic medium [36]. Therefore, we used AO to stain the acidic compartment, if any, of the cells treated with YRL1091. Fluorescence images revealed that YRL1091-treated cells did not show any orange fluorescence, indicating no acidic compartment in the YRL1091-treated cells (Figure 6F). Conversely, the cells challenged with Rapa, a well-known autophagy inducer, exhibited a prominent enhancement of the orange fluorescence (Figure 6F). Based on these observations, the participation of lysosomes in the YRL1091-induced vacuolization can be excluded. Collectively, our data confirmed that cytoplasmic vacuolization and cell death triggered by YRL1091 were not autophagic in nature.

#### 3.3.2. Non-Apoptotic Characteristics

**Figure 7:**
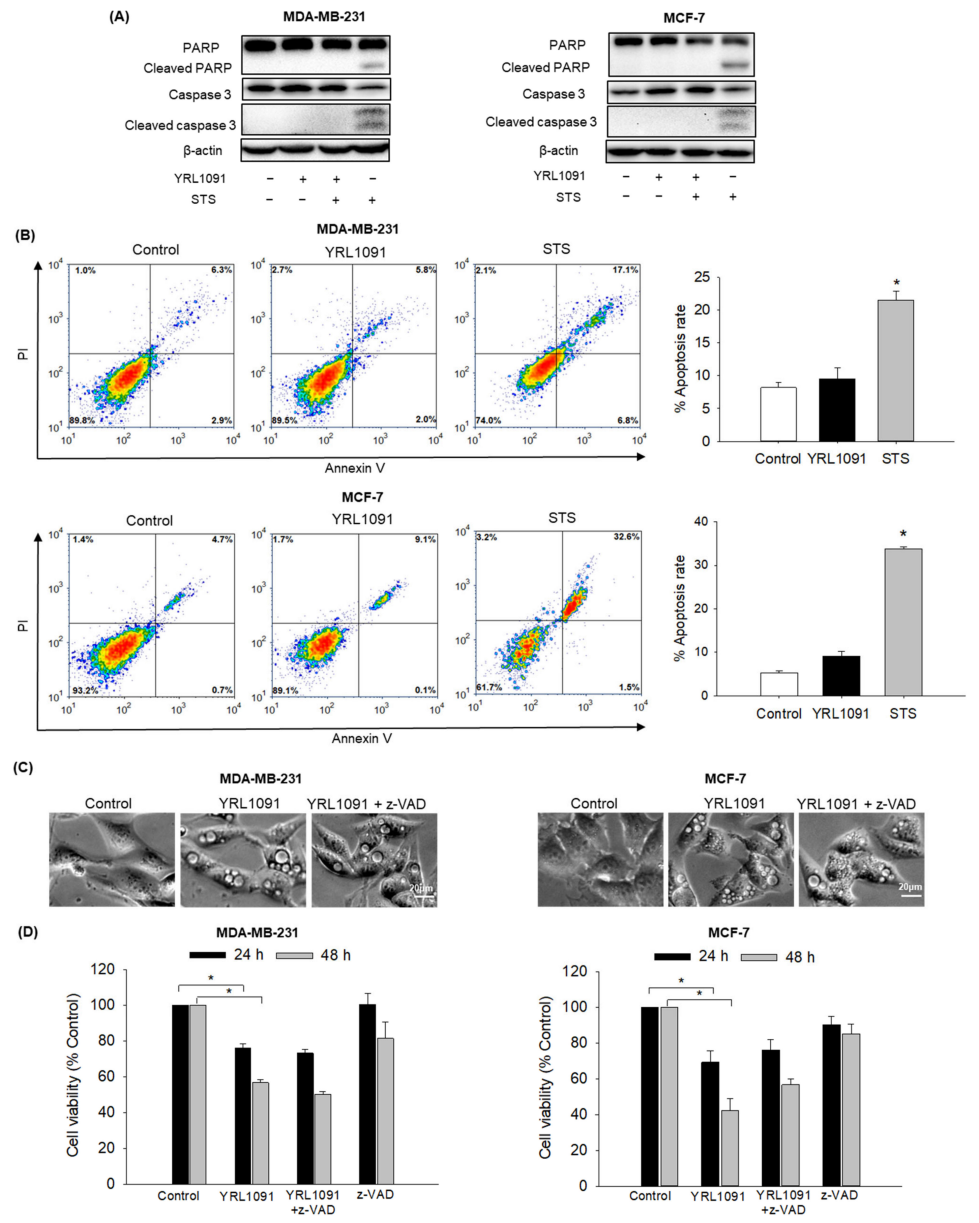
Apoptosis-independent features of YRL1091-treated MDA-MB-231 and MCF-7 cells. (**A**,**B**) Cells were treated with 30 µM YRL1091 for 24 h or 0.1 µM STS for 12 h. (**A**) Levels of cleaved PARP, PARP, cleaved caspase 3, and caspase 3 were determined by immunoblotting. (**B**) Annexin V/PI double-staining was carried out to determine the percentages of apoptotic cells. Representative scatter plots from flow cytometry were presented. (**C**,**D**) Cells were pretreated with or without 10 µM z-VAD for 2 h, followed by the addition of 30 µM YRL1091. (**C**) Cytoplasmic vacuoles were observed after 12 h of treatment. Scale bar: 20 µm. (**D**) Cell viability at 24 and 48 h was assessed by SRB assay. The cell viability of each treated group was expressed as the percentage of that in control group. Data are presented as the mean ± SEM from at least three independent experiments. * *p* < 0.05 vs. vehicle-treated control cells.

To investigate whether apoptosis was also involved in YRL1091-induced cell death in MDA-MB-231 and MCF-7 cells, the activations of PARP and caspase 3 were assessed as apoptotic markers in YRL1091-treated cells. STS was included as a positive control to induce apoptosis. As illustrated in Figure 7A, cleaved forms of PARP and caspase 3 were not detected in YRL1091-treated cells. By contrast, STS led to marked increases in the cleaved PARP and caspase 3 (Figure 7A). To further confirm that YRL1091-induced cell death was independent of apoptosis, annexin V/PI double-staining was performed. Using flow cytometry, we found that YRL1091 did not significantly change the percentage of apoptotic cells, whereas STS resulted in a dramatic increase in this parameter (Figure 7B). Furthermore, we tested the effect of a potent caspase inhibitor z-VAD on the cytoplasmic vacuoles and cytotoxicity induced by YRL1091. Despite the presence of z-VAD, the appearance of cytoplasmic vacuolization (Figure 7C) and cell death (Figure 7D) were not significantly altered. Altogether, these results indicated that the vacuolization and cytotoxic effects of YRL1091 in MDA-MB-231 and MCF-7 cells were not apoptotic in nature.

### 3.4. Generation of Intracellular ROS Contributes to YRL1091-Induced Cell Death and Cytoplasmic Vacuolization

**Figure 8:**
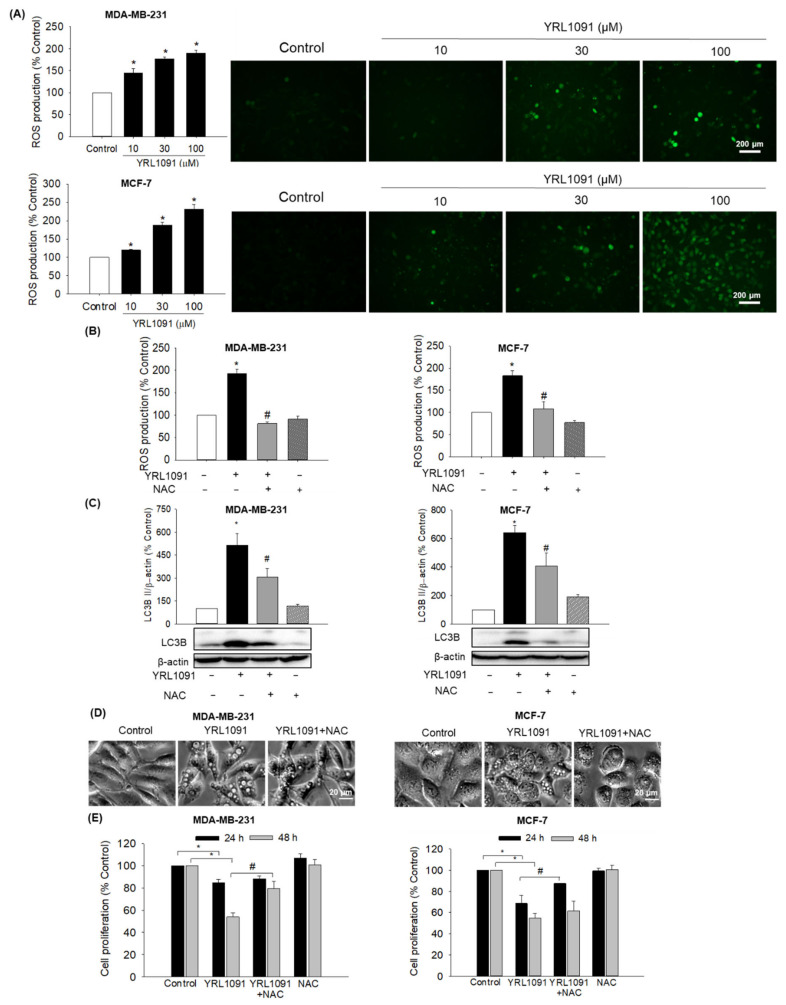
Involvement of YRL1091-induced ROS production in cytoplasmic vacuolization and cell death in MDA-MB-231 and MCF-7 cells. (**A**) Cells were challenged with YRL1091 at 10, 30, and 100 µM for 12 h. Intracellular ROS levels were quantified by DCFH-DA assay as described in Materials and Methods. Representative fluorescence microscopic images were shown. (**B**) Cells were pretreated with 10 mM NAC for 2 h, followed by exposure to 30 µM YRL1091 for 12 h. Intracellular ROS levels were caculated as the pecentages of those mesured in vehicle-treated control cells. (**C**) Cells were treated as described in (**B**) for 24 h and subjected to western blotting for LC3B. Each data point was calculated as the percentage of protein level in vehicle-treated control cells. Representative blots were presented. (**D**) Phase-contrast images were obtained after 12 h of treatment. Scale bar: 20 µm. (**E**) Cell viability was determined by SRB assay at 24 and 48 h. The cell viability of each treated group was expressed as the percentage of that in control group. Data are displayed as the mean ± SEM from at least three independent experiments. * *p* < 0.05 vs. vehicle-treated control cells; # *p* < 0.05 vs. YRL1091-treated cells.

Generation of ROS in malignant cells is acknowledged to trigger signaling cascades of cell death, including paraptosis [37,38]. We thus evaluated whether YRL1091 augmented the ROS production in MDA-MB-231 and MCF-7 cells, using DCFH-DA as a probe. We observed that the cells treated with YRL1091 concentration-dependently enhanced the generation of intracellular ROS, which was further ascertained by fluorescence microscopy (Figure 8A) in both types of BC cells. To determine the roles of ROS production in YRL1091-induced cytoplasmic vacuolization and cell death, we pretreated the cells with NAC, a specific ROS inhibitor. As expected, NAC markedly abolished ROS production (Figure 8B), as well as LC3B in MDA-MB-231 and MCF-7 cells treated with YRL1091 (Figure 8C). The formation of vacuoles by YRL1091 was also markedly impaired by NAC in both cell types (Figure 8D). While the suppression of YRL1091-induced cell death by NAC was detectable at 24 h in MCF-7 cells, it took 48 h in MDA-MB-231 cells (Figure 8E). NAC might have an impact on YRL1091-suppressed cell viability, depending on the cell type. These data suggested that the induction of cytoplasmic vacuolization and cell death by YRL1091 was mediated by intracellular ROS generation.

### 3.5. YRL1091-Mediated Cell Death Requires Protein Synthesis

**Figure 9:**
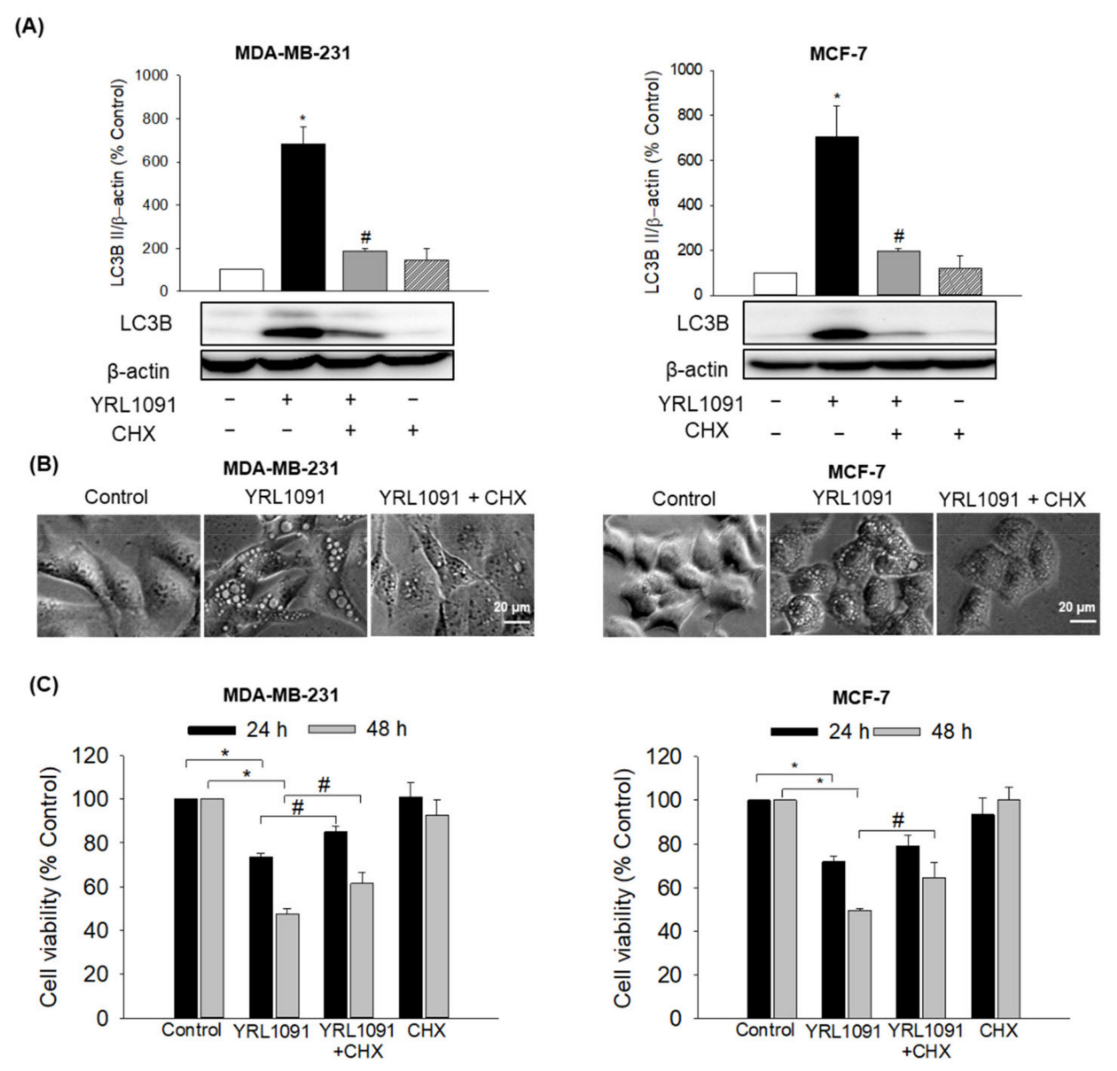
Requirement of protein synthesis in YRL1091-induced cytoplasmic vacuolization and cell death in MDA-MB-231 and MCF-7 cells. (**A**–**C**) Cells were treated with 30 µM YRL1091 in the presence or absence of CHX (2 µM, pretreatment for 2h) for 24 h. (**A**) Levels of LC3B were measured by western blotting. Each data point was calculated as the percentage of protein level in vehicle-treated control cells. Representative bands from at least three independent experiments were shown. (**B**) Phase-contrast images were captured. Scale bar: 20 µm. (**C**) Cell viability was determined by SRB assay at 24 and 48 h. The cell viability of each treated group was expressed as the percentage of that in control group. Data are presented as the mean ± SEM from at least three independent experiments.* *p* < 0.05 vs. vehicle-treated control cells; # *p* < 0.05 vs. YRL1091-treated cells.

Paraptotic cell death accompanied by the production of vacuoles is known to require new protein synthesis, which can be abrogated by CHX, a translation inhibitor [6,7]. It is worth validating whether the protein synthesis is required for the cytoplasmic vacuolization and cell death induced by YRL1091. Therefore, the effects of CHX on the expression of LC3B, vacuolization, and cell viability were measured in YRL1091-treated BC cells. We found that CHX dramatically inhibited the YRL1091-induced upregulation of LC3B and vacuole formation in both types of BC cells (Figure 9A and 9B, respectively). The cytotoxic effect of YRL1091 was significantly reversed by CHX in MDA-MB-231 cells at 24 h and maintained up to 48 h, while CHX significantly reversed the cytotoxicity in MCF-7 cells at 48 h only (Figure 9C). Taken together, our findings prove that YRL1091-induced paraptotic cell death in BC cells requires the synthesis of new proteins.

### 3.6. YRL1091-Induced Cytoplasmic Vacuoles Are Derived from ER Structure

**Figure 10:**
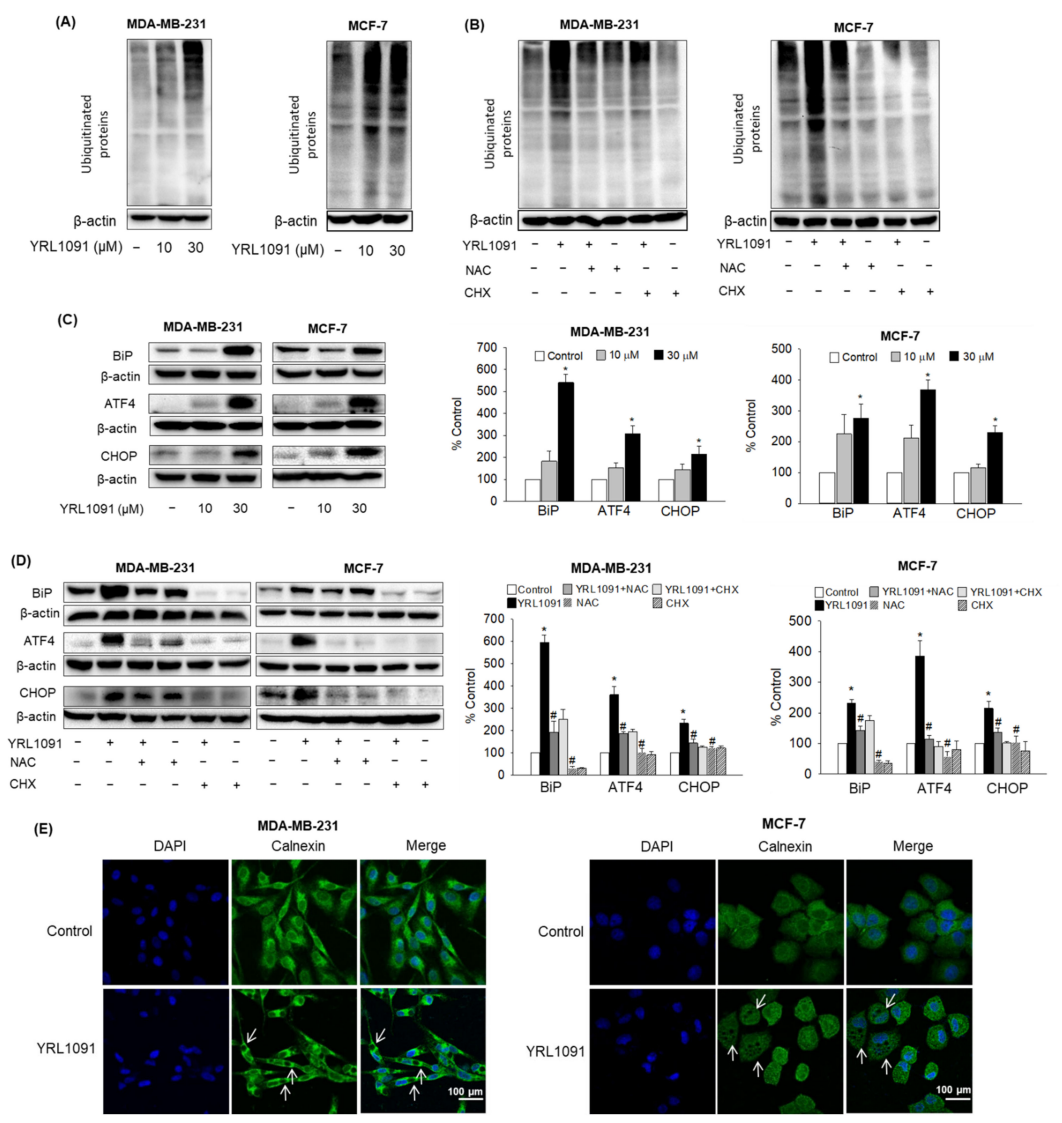
Induction of ER stress by YRL1091 and the origin of vacuoles in MDA-MB-231 and MCF-7 cells. (**A**,**C**) Cells were treated with YRL1091 (10 and 30 µM) for 24 h. (**B**,**D**), Cells were pretreated with 10 mM NAC or 2 µM CHX for 2 h, and then exposed to 30 µM YRL1091 for 24 h. After the desired treatments, immunoblotting analyses were performed using anti-poly-ubiquitinated protein antibody (**A**,**B**) or anti-BiP, -ATF4, and -CHOP antibodies (**C**,**D**). Each data point was calculated as the percentage of protein level in vehicle-treated control cells. Representative blots and relative levels of the respective protein from at least three independent experiments were displayed. Data are presented as the mean ± SEM from at least three independent experiments.* *p* < 0.05 vs. vehicle-treated control cells; # *p* < 0.05 vs. YRL1091-treated cells. (**E**) Cells were treated with 30 µM YRL1091 and stained with calnexin as described in Materials and Methods. Representative immunofluorescence images are shown. Scale bar: 100 μm. The white arrows indicate representative cytoplasmic vacuoles.

As described above, our study determined that the induction of paraptosis by YRL1091 was linked to the generation of intracellular ROS and required the synthesis of new proteins, which were known to cause ER stress. In addition, paraptosis displaying cytoplasmic vacuolization has been demonstrated to arise from ER stress in most cases [6,7]. To identify potential upstream signals mediating paraptosis, the effects of YRL1091 on ER stress were examined in MDA-MB-231 and MCF-7 cells. As a result, YRL1091 increased the levels of poly-ubiquitinated proteins in both types of BC cells (Figure 10A). Pretreatment with NAC or CHX followed by YRL1091 exposure successfully blocked the ubiquitinated protein accumulation (Figure 10B), indicating that ROS generation and protein synthesis are involved in the protein ubiquitination. The expressions of ER stress markers, including BiP, ATF4, and CHOP, were measured in both types of BC cells. All these proteins showed drastic increases in YRL1091-treated BC cells (Figure 10C), suggesting the development of ER stress by YRL1091. These marked enhancements were also suppressed by NAC or CHX (Figure 10D), suggesting the involvement of oxidative stress and unfolded proteins in ER stress caused by YRL1091.

Finally, we investigated the morphological perturbations in the ER by staining the cells with calnexin, an ER membrane-bound protein. Immunocytochemical analysis showed that, while calnexin was widely distributed in the cytoplasm of control-treated cells, it appeared to be localized at the membrane enclosing the vacuoles in YRL1091-treated cells (Figure 10E), indicating that cytoplasmic vacuoles formed by YRL1091 were derived from the ER structure in BC cells.

## 4. Discussion

In an attempt to discover potential anticancer agents with heterocyclic scaffolds, we synthesized a novel derivative of a pyrazolo[3,4-*h*]quinoline scaffold (YRL1091) and evaluated its potential anticancer effect in MDA-MB-231 and MCF-7 cells. YRL1091 exhibited cytotoxicity in these BC cells, which entailed the formation of numerous cytoplasmic vacuoles, a typical feature of paraptosis (Figure 1). Based on this observation, we further aimed to elucidate the molecular mechanisms of cell death mediating the anticancer effect of this compound. We found that paraptosis was the primary mode of PCD in both types of YRL1091-treated BC cells. This paraptotic PCD lacked apoptotic and autophagic features. The cells did not respond to typical inhibitors of caspase 3 or autophagy. Intracellular ROS generation and new protein synthesis were found to mediate ER stress, subsequently leading to cytoplasmic vacuolization and cell death. These findings suggest that YRL1091 may be a promising alternative candidate for BC treatment targeting paraptosis, a non-apoptotic form of cell death.

The formation of vacuoles often contributes to cell death, mainly due to the loss of membrane integrity and detachment from the substratum of cells [5]. The inducers of vacuolization cause well-known types of caspase-independent cell death, such as paraptosis, autophagy, methuosis, oncosis, and necrosis [5,39]. It is not easy to unambiguously and exclusively decipher these types of PCD, especially non-apoptotic PCD; however, a few surrogate indicators may aid to elucidate the mechanisms of cell death. We propose that the cell death mechanism triggered by YRL1091 appears to be paraptosis, not other forms of PCD, including autophagy and apoptosis. Firstly, vacuoles of varied sizes were observed, occupying nearly all of the cytoplasm of cells (Figure 1B). Newly formed blebs from the cell membrane and nuclear fragmentation were not detected, excluding the involvement of oncosis and necrosis [40,41]. We also proved the central role of LC3B protein in the formation of cytoplasmic vacuoles by YRL1091 in BC cells, as reported previously [42]. YRL1091 upregulated the level of LC3B in time- and concentration-dependent manners (Figure 2A), which was confirmed by immunocytochemistry (Figure 2B). LC3B depletion with siLC3B transfection (Figure 2C) blocked the formation of extensive cytoplasmic vacuoles and cell death caused by YRL1091 (Figure 2D,E). However, 3-MA, an inhibitor of autophagosome formation [43], did not alter YRL1091-induced upregulation of LC3B (Figure 6B), cytoplasmic vacuolization (Figure 6E), and cell death (Figure 6F). In parallel with previous reports that LC3B induction is associated with autophagy-independent mechanisms [31,44,45], our findings demonstrate the vital role of LC3B in YRL1091-induced paraptosis.

In methuosis, LC3 is not localized in the vacuole membranes [5], so the cytotoxic effect of YRL1091 did not involve methuosis. Furthermore, paraptotic PCD is shown to activate insulin-like growth factor I receptor (IGF-IR), thereby regulating MAPKs, including ERK1/2, JNK, and p38 MAPK [5]. Consistent with this previous findings, phosphorylated ERK1/2 was significantly increased in both YRL1091-treated MDA-MB-231 and MCF-7 cells. However, phosphorylated JNK was markedly increased only in YRL1091-treated MCF-7 cells (Figure 3A,B). Additionally, the inhibition of ERK1/2 by U0126 markedly suppressed YRL1091-induced LC3B expression, cytoplasmic vacuolization, and cell death in both MDA-MB-231 and MCF-7 cells (Figure 3C–E). Interestingly, the JNK inhibitor, SP600125 did not influence the protein level of LC3B in MCF-7 cells; however, it could suppress the cytotoxic effect and vacuolization induced by YRL1091 (Figure 3C–E). There may be a distinct, cell type-specific mechanism underlying YRL1091-induced paraptosis in MCF-7 cells that is mediated by JNK without affecting LC3B. Further studies are required to prove this possibility.

IGF-IR-mediated paraptosis is reported to be blocked by AIP-1/Alix protein, which helps to distinguish paraptosis from apoptosis [33]. Our data revealed a reduction of Alix protein (Figure 4), indicating the participation of paraptosis-mediated cell death by YRL1091. The cell type-specific effect of YRL1091 is also represented in the activation of caspase 9. Although paraptosis is a caspase-independent PCD, the activation of caspase 9 has been reported to be associated with paraptosis [46]. In our study, the cleaved form of caspase 9 was only detected in MDA-MB-231 cells, not in MCF-7 cells (Figure 5). The significance of this difference may require further study.

The mode of cell death mediated by YRL1091 was proved to be non-autophagic. In addition to LC3B, p62 protein, a predictor of autophagic flux, was significantly augmented in YRL1091-treated cells with increased concentration and exposure time (Figure 6A), which was not affected by autophagy inhibitors (Figure 6C). Furthermore, YRL1091 caused cell death and vacuoles even in the presence of 3-MA or CQ (Figure 6D,E). Since p62 is thoroughly explored to be degraded at the later stage of autophagy [47], these data further support excluding the involvement of autophagy in YRL1091-treated BC cells. Moreover, YRL1091-induced vacuoles tracked by AO staining did not contain acidic vesicular organelles, in contrast to the vacuoles in autophagy (Figure 6F). Therefore, our results demonstrate the correlation of paraptotic vacuoles with the accumulation of p62 and non-acidic compartments, commensurate with previous studies on paraptosis [44,45,48].

Apoptosis is the key mechanism by which chemotherapeutic treatment kills tumor cells. Generally, paraptosis occurs concurrently with apoptosis induction [46]. We continued to explore whether the mode of cell death triggered by YRL1091 involved apoptosis. We found that the cytotoxic effect of YRL1091 was caspase-independent because there was no cleavages of PARP and caspase 3 (Figure 7A). Moreover, only a minor percentage of cells were positive for apoptosis in annexin V/PI double-staining (Figure 7B). Furthermore, YRL1091-induced cytoplasmic vacuolization and cell death were not abrogated by a caspase inhibitor (Figure 7C,D), differentiating the effect of YRL1091 from the conventional apoptotic pathway. Collectively, our aforementioned data showed that paraptotic cell death was the primary mode of PCD in YRL1091-treated BC cells.

It is well established that the escalated ROS production contributes to mitochondrial dysfunction and/or ER stress, which is a prerequisite for the formation of paraptotic vacuoles [6,7]. However, excess ROS are often quenched by increased antioxidant enzymatic and nonenzymatic pathways in cancer cells, eventually leading tumor development and drug resistance [10,37,49]. We found that ROS levels were concentration-dependently elevated in both MDA-MB-231 and MCF-7 cells challenged with YRL1091 (Figure 8A), which were subsequently inhibited by NAC (Figure 8B). Furthermore, NAC also suppressed the upregulated LC3B, vacuole formation, and cell death caused by YRL1091 (Figure 8C–E), confirming that the YRL1091-induced ROS generation mediated paraptotic phenotype in BC cells. Intriguingly, the suppressive effects of NAC in YRL1091-mediated cell death may be dependent on cell type since different exposure times are required for the cells to recover (Figure 8E). Further investigations to decipher the meaning of this discrepancy may lead to a better understanding of the anticancer effect of YRL1091 in different types of BC cells.

Protein synthesis is one of the vital cellular processes that facilitate tumor growth. However, uncontrollable upregulation of protein synthesis can be harmful to cells since it raises cellular stress [50]. The accumulation of misfolded and/or unfolded proteins that cause ER stress has been linked to paraptotic-like cell death [6,7]. Hence, CHX, a protein synthesis inhibitor, can block cell death by easing the burden on the homeostatic protein-folding machinery [51]. In our study, the increased expression of LC3B, the vacuolated cells, as well as cell death executed by YRL1091 were potently impeded by CHX (Figure 9), suggesting the requirement for new protein synthesis in YRL1091-induced cytotoxicity. This reliance on protein synthesis emphasizes the programmed nature of paraptotic form.

Folding-defective proteins are recognized by ER-associated protein degradation, which are then translocated to the cytoplasm and degraded by the ubiquitin-proteasome system [52,53]. In rapidly proliferating tumor cells, the imbalance between a high metabolic rate and a limited protein folding capacity often leads to overloaded ER with unfolded and misfolded proteins [54]. Dysfunctional ER concurrent with paraptotic vacuolization has been extensively studied previously [48,55,56]. To gain further insight into the mechanisms underlying the anticancer activity of YRL1091, we continued to investigate whether YRL1091-induced vacuoles were arisen from the ER. We observed that YRL1091 effectively induced the accumulations of ubiquitinated proteins in MDA-MB-231 and MCF-7 cells (Figure 10A), which were potently blocked by CHX (Figure 10B), suggesting that the ER appeared to be overwhelmed with misfolded proteins upon YRL1091 treatment. Accumulation of misfolded proteins activates BiP, which acts as a chaperone in the ER lumen, stimulating three major sensors to cope with ER stress. One of these protein sensors, protein kinase RNA-like ER kinase, allows preferential translation of the ER stress-induced transcription factor ATF4, which then mediates the induction of pro-death transcriptional regulator, CHOP [57,58]. Our data showed increased levels of BiP, ATF4, and CHOP in YRL1091-treated BC cells (Figure 10C). Moreover, CHX, a well-known inhibitor of dilation of cellular organelles including the ER, potently reversed these enhancements (Figure 10D). One of the most prevalent morphological characteristics generated by paraptosis is ER dilation [51], reinforcing the association between the activity of YRL1091 with the biochemical features of paraptosis. In addition to the determination of the key proteins in the ER stress response, we also noted perturbations in the morphology of the ER in the calnexin-stained YRL1091-treated cells. Our findings suggest that the multiple cytoplasmic vacuoles triggered by YRL1091 were derived from the ER structures (Figure 10E). The production of ROS also increases the amount of unfolded proteins in the ER, resulting in the ER stress responses [59]. In our findings, NAC drastically alleviated the ubiquitinated protein accumulation, and the expressions of ER stress markers, including BiP, ATF4, and CHOP (Figure 10B,D), indicating an upstream role for ROS in the induction of ER stress targeting paraptosis.

**Figure 11:**
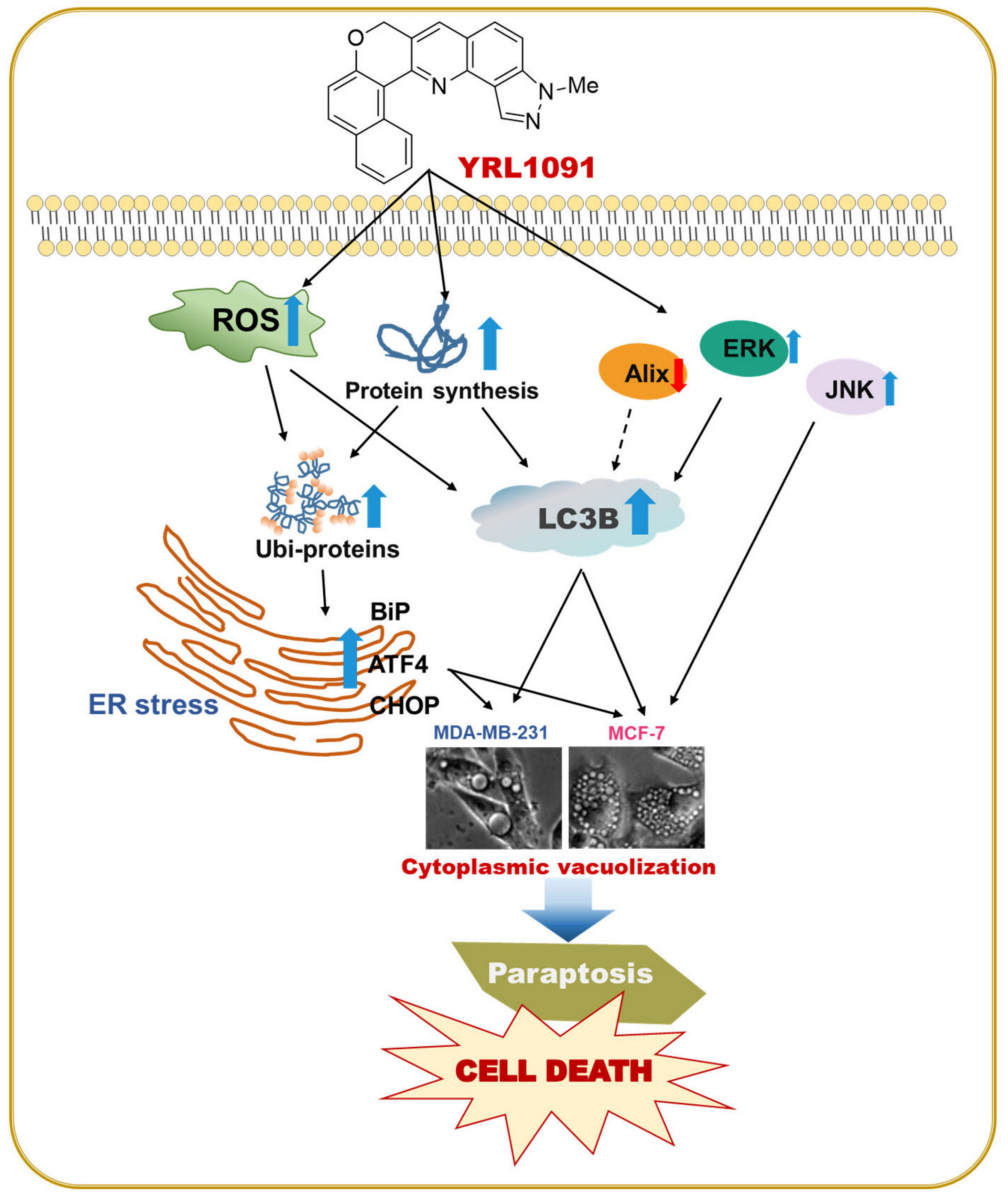
Schematic illustration of the molecular mechanisms of cell death triggered by YRL1091, leading to paraptotic cytotoxicity in MDA-MB-231 and MCF-7 breast cancer cells. The black arrows indicate pathways examined in this study, while the black broken arrow shows a pathway not examined yet. ROS, reactive oxygen species; ER, endoplasmic reticulum; BiP, binding immunoglobulin protein; ATF4, activating transcription factor-4; CHOP, CCAAT-enhancer-binding protein homologous protein; LC3B, microtubule-associated protein 1 light chain 3B; Alix, multifunctional adapter protein; ERK1/2, extracellular signal-regulated kinase 1/2; JNK, c-Jun N-terminal kinase.

A schematic illustration depicting the proposed mechanisms of YRL1091-induced cytotoxicity in conjunction with cytoplasmic vacuolization in MDA-MB-231 and MCF-7 cells is shown (Figure 11). In brief, YRL1091-induced paraptotic cell death was closely correlated with the upregulation of LC3B, the suppression of Alix protein, and the activation MAPKs, including ERK 1/2 and JNK. It also increased the production of ROS and required new protein synthesis, subsequently causing ubiquitinated protein accumulation and ER stress. Taken together, YRL1091 is an effective inducer of paraptosis, which may represent a novel alternative agent for BC treatment to overcome chemoresistant BC.

## 5. Conclusions

In conclusion, here we report for the first time the anticancer effect of a novel synthetic derivative of a heterocyclic scaffold of pyrazolo[3,4-*h*]quinoline (YRL1091) and its underlying molecular mechanisms of cell death using MDA-MB-231 and MCF-7 BC cells. Several key features, including elevated expression of LC3B, decreased level of Alix, and activation of ERK1/2 and JNK, strongly support that YRL1091 triggers paraptotic PCD in both types of BC cells. Our findings also reveal that cytoplasmic vacuolization and cytotoxicity triggered by YRL1091 are not autophagic or apoptotic in nature. Furthermore, YRL1091-induced paraptosis requires newly synthesized proteins and ROS production, which mediates ER stress, subsequently leading to extensive cytoplasmic vacuolization. Based on our findings, YRL1091 may serve as a novel potential anticancer agent triggering paraptosis, therein providing complementary or alternative approaches to treat BC resistant to conventional chemotherapy.

## Data Availability

All of the data are contained within the article.

## References

[1] Sung H., Ferlay J., Siegel R.L., Laversanne M., Soerjomataram I., Jemal A., Bray F. (2021). Global Cancer Statistics 2020: GLOBOCAN Estimates of Incidence and Mortality Worldwide for 36 Cancers in 185 Countries. CA Cancer J. Clin..

[2] Byler S., Goldgar S., Heerboth S., Leary M., Housman G., Moulton K., Sarkar S. (2014). Genetic and epigenetic aspects of breast cancer progression and therapy. Anticancer Res..

[3] Luque-Bolivar A., Pérez-Mora E., Villegas V.E., Rondón-Lagos M. (2020). Resistance and Overcoming Resistance in Breast Cancer. Breast Cancer.

[4] Khalili M., Radosevich J.A., Radosevich J.A. (2018). Paraptosis. Apoptosis and Beyond.

[5] Shubin A.V., Demidyuk I.V., Komissarov A.A., Rafieva L.M., Kostrov S.V. (2016). Cytoplasmic vacuolization in cell death and survival. Oncotarget.

[6] Fontana F., Raimondi M., Marzagalli M., Di Domizio A., Limonta P. (2020). The emerging role of paraptosis in tumor cell biology: Perspectives for cancer prevention and therapy with natural compounds. Biochim Biophys Acta Rev. Cancer.

[7] Wang Y., Wen X., Zhang N., Wang L., Hao D., Jiang X., He G. (2019). Small-molecule compounds target paraptosis to improve cancer therapy. Biomed Pharmacother.

[8] Yoon M.J., Kim E.H., Lim J.H., Kwon T.K., Choi K.S. (2010). Superoxide anion and proteasomal dysfunction contribute to curcumin-induced paraptosis of malignant breast cancer cells. Free Radic. Biol. Med..

[9] Yoon M.J., Lee A.R., Jeong S.A., Kim Y.S., Kim J.Y., Kwon Y.J., Choi K.S. (2014). Release of Ca2+ from the endoplasmic reticulum and its subsequent influx into mitochondria trigger celastrol-induced paraptosis in cancer cells. Oncotarget.

[10] Ghosh K., De S., Das S., Mukherjee S., Sengupta Bandyopadhyay S. (2016). Withaferin A Induces ROS-Mediated Paraptosis in Human Breast Cancer Cell-Lines MCF-7 and MDA-MB-231. PLoS ONE.

[11] Rolver M.G., Elingaard-Larsen L.O., Andersen A.P., Counillon L., Pedersen S.F. (2020). Pyrazine ring-based Na(+)/H(+) exchanger (NHE) inhibitors potently inhibit cancer cell growth in 3D culture, independent of NHE1. Sci. Rep..

[12] Dam J., Ismail Z., Kurebwa T., Gangat N., Harmse L., Marques H.M., Lemmerer A., Bode M.L., de Koning C.B. (2017). Synthesis of copper and zinc 2-(pyridin-2-yl)imidazo[1,2-a]pyridine complexes and their potential anticancer activity. Eur. J. Med. Chem..

[13] Gaurav A., Gautam V., Singh R. (2010). An overview on synthetic methodologies and biological activities of pyrazoloquinolines. Mini Rev. Med. Chem..

[14] Opoku-Temeng C., Dayal N., Aflaki Sooreshjani M., Sintim H.O. (2018). 3H-pyrazolo[4,3-f]quinoline haspin kinase inhibitors and anticancer properties. Bioorganic Chem..

[15] Spanò V., Parrino B., Carbone A., Montalbano A., Salvador A., Brun P., Vedaldi D., Diana P., Cirrincione G., Barraja P. (2015). Pyrazolo[3,4-h]quinolines promising photosensitizing agents in the treatment of cancer. Eur. J. Med. Chem..

[16] Azizmohammadi M., Khoobi M., Ramazani A., Emami S., Zarrin A., Firuzi O., Miri R., Shafiee A. (2013). 2H-chromene derivatives bearing thiazolidine-2,4-dione, rhodanine or hydantoin moieties as potential anticancer agents. Eur. J. Med. Chem..

[17] Pratap R., Ram V.J. (2014). Natural and synthetic chromenes, fused chromenes, and versatility of dihydrobenzo[h]chromenes in organic synthesis. Chem. Rev..

[18] Choi M., Hwang Y.S., Kumar A.S., Jo H., Jeong Y., Oh Y., Lee J., Yun J., Kim Y., Han S.B. (2014). Design and synthesis of 3,4-dihydro-2H-benzo[h]chromene derivatives as potential NF-κB inhibitors. Bioorg. Med. Chem. Lett..

[19] Sim S., Lee S., Ko S., Phuong Bui B., Linh Nguyen P., Cho J., Lee K., Kang J.-S., Jung J.-K., Lee H. (2021). Design, synthesis, and biological evaluation of potent 1,2,3,4-tetrahydroisoquinoline derivatives as anticancer agents targeting NF-κB signaling pathway. Bioorg. Med. Chem..

[20] Arepalli S.K., Lee C., Sim S., Lee K., Jo H., Jun K.Y., Kwon Y., Kang J.S., Jung J.K., Lee H. (2018). Development of 13H-benzo[f]chromeno[4,3-b][1,7]naphthyridines and their salts as potent cytotoxic agents and topoisomerase I/IIα inhibitors. Bioorg. Med. Chem..

[21] Huang Z., Yu P., Tang J. (2020). Characterization of Triple-Negative Breast Cancer MDA-MB-231 Cell Spheroid Model. Onco. Targets Ther..

[22] Collignon J., Lousberg L., Schroeder H., Jerusalem G. (2016). Triple-negative breast cancer: Treatment challenges and solutions. Breast Cancer.

[23] Comşa Ş., Cîmpean A.M., Raica M. (2015). The Story of MCF-7 Breast Cancer Cell Line: 40 years of Experience in Research. Anticancer Res..

[24] Do H.T.T., Cho J. (2020). Involvement of the ERK/HIF-1α/EMT Pathway in XCL1-Induced Migration of MDA-MB-231 and SK-BR-3 Breast Cancer Cells. Int. J. Mol. Sci..

[25] Lee J.Y., Park M.K., Park J.H., Lee H.J., Shin D.H., Kang Y., Lee C.H., Kong G. (2014). Loss of the polycomb protein Mel-18 enhances the epithelial-mesenchymal transition by ZEB1 and ZEB2 expression through the downregulation of miR-205 in breast cancer. Oncogene.

[26] Orellana E.A., Kasinski A.L. (2016). Sulforhodamine B (SRB) Assay in Cell Culture to Investigate Cell Proliferation. Bio-protocol.

[27] Moniruzzaman M., Bose S., Kim Y.M., Chin Y.W., Cho J. (2016). The ethyl acetate fraction from Physalis alkekengi inhibits LPS-induced pro-inflammatory mediators in BV2 cells and inflammatory pain in mice. J. Ethnopharmacol..

[28] Nguyen P.L., Bui B.P., Lee H., Cho J. (2021). A Novel 1,8-Naphthyridine-2-Carboxamide Derivative Attenuates Inflammatory Responses and Cell Migration in LPS-Treated BV2 Cells via the Suppression of ROS Generation and TLR4/Myd88/NF-κB Signaling Pathway. Int. J. Mol. Sci..

[29] Nguyen P.L., Bui B.P., Duong M.T.H., Lee K., Ahn H.C., Cho J. (2021). Suppression of LPS-Induced Inflammation and Cell Migration by Azelastine through Inhibition of JNK/NF-κB Pathway in BV2 Microglial Cells. Int. J. Mol. Sci..

[30] Wang W.B., Feng L.X., Yue Q.X., Wu W.Y., Guan S.H., Jiang B.H., Yang M., Liu X., Guo D.A. (2012). Paraptosis accompanied by autophagy and apoptosis was induced by celastrol, a natural compound with influence on proteasome, ER stress and Hsp90. J. Cell Physiol..

[31] Li X.Q., Ren J., Wang Y., Su J.Y., Zhu Y.M., Chen C.G., Long W.G., Jiang Q., Li J. (2021). Synergistic killing effect of paclitaxel and honokiol in non-small cell lung cancer cells through paraptosis induction. Cell Oncol..

[32] Kar R., Singha P.K., Venkatachalam M.A., Saikumar P. (2009). A novel role for MAP1 LC3 in nonautophagic cytoplasmic vacuolation death of cancer cells. Oncogene.

[33] Sperandio S., Poksay K., de Belle I., Lafuente M.J., Liu B., Nasir J., Bredesen D.E. (2004). Paraptosis: Mediation by MAP kinases and inhibition by AIP-1/Alix. Cell Death Differ..

[34] González-Polo R.A., Boya P., Pauleau A.L., Jalil A., Larochette N., Souquère S., Eskelinen E.L., Pierron G., Saftig P., Kroemer G. (2005). The apoptosis/autophagy paradox: Autophagic vacuolization before apoptotic death. J. Cell. Sci..

[35] Jiang P., Mizushima N. (2015). LC3- and p62-based biochemical methods for the analysis of autophagy progression in mammalian cells. Methods.

[36] Krolenko S.A., Adamyan S.Y., Belyaeva T.N., Mozhenok T.P. (2006). Acridine orange accumulation in acid organelles of normal and vacuolated frog skeletal muscle fibres. Cell Biol. Int..

[37] Chen X., Chen X., Zhang X., Wang L., Cao P., Rajamanickam V., Wu C., Zhou H., Cai Y., Liang G. (2019). Curcuminoid B63 induces ROS-mediated paraptosis-like cell death by targeting TrxR1 in gastric cells. Redox Biol..

[38] Shiau J.Y., Nakagawa-Goto K., Lee K.H., Shyur L.F. (2017). Phytoagent deoxyelephantopin derivative inhibits triple negative breast cancer cell activity by inducing oxidative stress-mediated paraptosis-like cell death. Oncotarget.

[39] Kessel D. (2019). Apoptosis, Paraptosis and Autophagy: Death and Survival Pathways Associated with Photodynamic Therapy. Photochem. Photobiol..

[40] Weerasinghe P., Buja L.M. (2012). Oncosis: An important non-apoptotic mode of cell death. Exp. Mol. Pathol..

[41] Hitomi J., Christofferson D.E., Ng A., Yao J., Degterev A., Xavier R.J., Yuan J. (2008). Identification of a molecular signaling network that regulates a cellular necrotic cell death pathway. Cell.

[42] Runwal G., Stamatakou E., Siddiqi F.H., Puri C., Zhu Y., Rubinsztein D.C. (2019). LC3-positive structures are prominent in autophagy-deficient cells. Sci. Rep..

[43] Ma Y., Hayat M.A. (2016). Chapter 13—Role of Autophagy in Cancer Therapy. Autophagy: Cancer, Other Pathologies, Inflammation, Immunity, Infection, and Aging.

[44] Nedungadi D., Binoy A., Pandurangan N., Pal S., Nair B.G., Mishra N. (2018). 6-Shogaol induces caspase-independent paraptosis in cancer cells via proteasomal inhibition. Exp. Cell Res..

[45] Binoy A., Nedungadi D., Katiyar N., Bose C., Shankarappa S.A., Nair B.G., Mishra N. (2019). Plumbagin induces paraptosis in cancer cells by disrupting the sulfhydryl homeostasis and proteasomal function. Chem. Biol. Interact..

[46] Sperandio S., de Belle I., Bredesen D.E. (2000). An alternative, nonapoptotic form of programmed cell death. Proc. Natl. Acad. Sci. USA.

[47] Liu W.J., Ye L., Huang W.F., Guo L.J., Xu Z.G., Wu H.L., Yang C., Liu H.F. (2016). p62 links the autophagy pathway and the ubiqutin-proteasome system upon ubiquitinated protein degradation. Cell Mol. Biol. Lett..

[48] Ram B.M., Ramakrishna G. (2014). Endoplasmic reticulum vacuolation and unfolded protein response leading to paraptosis like cell death in cyclosporine A treated cancer cervix cells is mediated by cyclophilin B inhibition. Biochim. Biophys. Acta.

[49] Sang J., Li W., Diao H.J., Fan R.Z., Huang J.L., Gan L., Zou M.F., Tang G.H., Yin S. (2021). Jolkinolide B targets thioredoxin and glutathione systems to induce ROS-mediated paraptosis and apoptosis in bladder cancer cells. Cancer Lett..

[50] Nguyen H.G., Conn C.S., Kye Y., Xue L., Forester C.M., Cowan J.E., Hsieh A.C., Cunningham J.T., Truillet C., Tameire F. (2018). Development of a stress response therapy targeting aggressive prostate cancer. Sci. Transl. Med..

[51] Lee D., Kim I.Y., Saha S., Choi K.S. (2016). Paraptosis in the anti-cancer arsenal of natural products. Pharmacol. Ther..

[52] Olzmann J.A., Kopito R.R., Christianson J.C. (2013). The mammalian endoplasmic reticulum-associated degradation system. Cold Spring Harb. Perspect. Biol..

[53] Lemus L., Goder V. (2014). Regulation of Endoplasmic Reticulum-Associated Protein Degradation (ERAD) by Ubiquitin. Cells.

[54] Suh D.H., Kim M.K., Kim H.S., Chung H.H., Song Y.S. (2012). Unfolded protein response to autophagy as a promising druggable target for anticancer therapy. Ann. N.Y. Acad. Sci..

[55] Mimnaugh E.G., Xu W., Vos M., Yuan X., Isaacs J.S., Bisht K.S., Gius D., Neckers L. (2004). Simultaneous inhibition of hsp 90 and the proteasome promotes protein ubiquitination, causes endoplasmic reticulum-derived cytosolic vacuolization, and enhances antitumor activity. Mol. Cancer Ther..

[56] Fontana F., Moretti R.M., Raimondi M., Marzagalli M., Beretta G., Procacci P., Sartori P., Montagnani Marelli M., Limonta P. (2019). δ-Tocotrienol induces apoptosis, involving endoplasmic reticulum stress and autophagy, and paraptosis in prostate cancer cells. Cell Prolif..

[57] Kennedy D., Samali A., Jäger R. (2015). Methods for studying ER stress and UPR markers in human cells. Methods Mol. Biol..

[58] Almanza A., Carlesso A., Chintha C., Creedican S., Doultsinos D., Leuzzi B., Luís A., McCarthy N., Montibeller L., More S. (2019). Endoplasmic reticulum stress signalling—from basic mechanisms to clinical applications. FEBS J..

[59] Farooqi A.A., Li K.T., Fayyaz S., Chang Y.T., Ismail M., Liaw C.C., Yuan S.S., Tang J.Y., Chang H.W. (2015). Anticancer drugs for the modulation of endoplasmic reticulum stress and oxidative stress. Tumour Biol..

